# Our New York Letter

**Published:** 1891-05

**Authors:** Wm. L. Russell

**Affiliations:** 151 East 50th Street; New York


### OUR NEW YORK LETTER.

NEW YORK, April 15, 1891.

This city has again been invaded by la grippe, which we hoped to have seen the last of a year ago. During the past few weeks the death rate here, in Brooklyn, and in several other northern cities has been markedly increased. The clinical history of the disease has varied in some respects from that of last year. Intense pain in the frontal region, with almost complete obstruction of the nasal passages was a prominent feature of many of the early cases; at present there is a decided tendency to the development of pneumonia, which is often of a peculiar, transient type. Nothing new has yet been discovered regarding the real nature of the disease, nor can it be said that any uniformly satisfactory treatment has been established.

### THE TREATMENT OF LUPUS.

At the last meeting of the Academy, this was the subject under consideration. The paper of the evening was read by Dr. Piffard, the well-known dermatologist. He stated that at one time lupus was confounded with cancer. It was then regarded as a manifestation of scrofula, and now it was believed to be a local tuberculosis. Many varieties of treatment had been recommended since the earliest times. The induction of a severe local inflammation by the application of an irritant was a method long in vogue. Biniodide of mercury was the agent usually employed; but the remedy was liable to cause injury, and generally proved too mild a stimulant, so that its use had been discarded. Boring with a sharp point of silver nitrate was still resorted to by many; but it was inefficient, as many outlying cells were sure to escape. Absolute destruction of every particle of diseased tissue was essential to the production of a cure. Excision, if practicable, would seem to promise the best results. It could only be employed, however, when the disease was distinctly circumscribed, and not more than an inch or so in diameter. Relapse was more frequent than after operations for epithelioma, as it was more difficult to remove every diseased point. Treatment by the actual cautery alone was seldom employed, and was not often successful. Curetting alone was also unsatisfactory. A combination of curetting and the cautery was, however, about as valuable a plan of treatment known. The cautery should be heated to redness only. A useful procedure was scarification with a platinum knife heated to redness by means of electricity. Repeated operations were usually necessary. An anæsthetic was not always required; in any case ether must not be used, because of the danger of explosion; nitrous oxide gas and opium answered every purpose.

In regard to the Koch treatment, it might be said that the statements of its originator had not been verified. Many men not especially familiar with skin diseases had discharged cases of lupus as "cured." To the eye of the specialist, however, they did not appear so, for the significant apple jelly granules could always be detected either immediately, or very soon after treatment had been discontinued. The cicatrix must appear completely colorless and blanched before it could be pronounced healthy. The action of Koch's fluid was certainly unique. It was unlike the action of such poisons as arsenic, strychnine, or atropine, which was similiar in the sick and the well. It was more like that of erysipelas or typhoid fever.

Dr. H. N. Heineman said that of the nine cases treated by the Koch method at Mt. Sinai Hospital, two had been pronounced cured, no relapse being noticeable seven weeks after treatment was discontinued. He believed that a combination of local and injection treatment would eventually prove of most value.

Dr. H. P. Loomis gave an account of a few cases treated at Bellevue Hospital. Of these only one was cured. Another, which was very severe, was improved so much after a short course of treatment, as to demonstrate very conclusively the great value of the remedy. Stereopticon views of these patients were exhibited, showing the different stages of the improvement under the action of the tuberculin. The most striking case was one of a very extensive lupus of the face and neck. After a short course of treatment, all the active ulceration ceased, and the general aspect of the disease was greatly changed.

Dr. R. W. Taylor did not think that the bacillary origin of lupus had been proven, nor had its association with pulmonary phthisis been generally noticed.

Dr. G. H. Fox was not in favor of treating any case by excision. He liked the curette better. The actual cautery or galvano-cautery was not so satisfactory as the potential caustics, which had a selective action. The curette and some agent producing suppurative inflammation, produced the most satisfactory results. He drew attention to the dental burr as a most effective instrument for destroying isolated tubercles. He had seen a number of cases treated by the Koch method, and regarded it as extremely valuable.

PREVENTION OF DIPHTHERIA.

The subject of diphtheria was again under consideration at the last meeting of the Academy Section in Pædiatrics, with especial regard to the question of prevention. Dr. A. Caillè, the chairman, opened the discussion. He stated that, during the year 1890, there had been 1,400 deaths from the disease in New York alone. He thought that much could be done to protect well children from contracting it. The early treatment of a naso-pharyngeal catarrh, and thorough cleansing of the mouth and teeth at all times, were points never to be neglected. He believed that the daily inspection of school children by physicians appointed by the city, was quite practicable, and would be the means of saving many lives.

Dr. J. Lewis Smith said that Welch, of Baltimore, had induced diphtheria in animals with cultures of the Klebs-Löffler bacillus. It could now be positively believed that this was the specific organism of the disease. The germ was possessed with marvellous vitality and could be conveyed in many different ways. Exposure in the room or to the breath of a sick person was often sufficient. The clothing of nurses of children who had been sick, or of physicians who, on examination of a throat, had been spattered with sputum, might be the vehicle of contagion. There were many walking cases, and the schools were often the means of bringing them in contact with the healthy. Prior to 1850, diphtheria did not prevail in New York. It then appeared, and the sewers became infected. Thus it happened that sewer gas produced the disease, and with the walking cases was the origin of most of the cases occurring here.

When a case developed in a family, all furniture which could be spared should be removed from the sick room, and only those engaged in caring for the patient should be permitted to enter. Ventilation should be carefully attended to, and the air of the room should be permeated by some antiseptic vapor. The convalescent patient should be carefully disinfected, and kept apart from other children at least a month. In the after disinfection of the room sulphur fumigation should be used, but was not entirely reliable. In addition all surfaces should be washed with corrosive sublimate solution.

Dr. Prudden, the pathologist, believed that it had become possible to make a diagnosis of diphtheria by means of biological cultures of the bacilli found. He advised the use of mild antiseptic mouth washes as a preventive measure.

Dr. Jacobi drew attention to the appalling fact that 40,000 children had died from diphtheria in this city alone during the past thirty years. In the face of such a fact the indifference of the public as compared with the excitment apparent in the press over a single case of typhus fever in Bellevue Hospital was astounding. One of the greatest necessities was a temporary home to which the well children of the poor could be taken when a contagious disease had invaded the home. The teachers of public schools should be taught to examine children's throats, and all suspected cases should be sent home.

Dr. L. Emmett Holt said that during the past four or five years there had not been an epidemic of diphtheria at the New

York Infant Asylum. There were four hundred infants at this institution nearly all under two years old, and more than half less than eighteen months. Sporadic cases appeared occasionally. As soon as they were observed each was isolated separately, and the ward from which they were taken was emptied. After fumigation with sulphur, all surfaces were washed with corrosive sublimate solution, and the iron bedsteads with a solution of carbolic acid. The throats of children who had been exposed were examined twice daily, and their nasal cavities syringed with 1 to 5000 bichloride solution.

### CRANIOTOMY *versus* CÆSAREAN SECTION.

Is embryotomy of the living fœtus ever justifiable? This was the question under discussion at the last meeting of the Section on Obstetrics at the Academy. Dr. E. H. Grandin read a paper advocating elective Cæsarean section. He believed that with a better knowledge of pelvimetry, and with the great improvements which have been of late years made in the operations, a deliberate choice of Cæsarean section would soon become the rule in cases in which the exact condition of affairs had been ascertained before other methods of delivery had been attempted and had failed. He thought the statistics of the operation misleading, as the majority of cases had been operated upon only as a last resort.

Dr. H. J. Garrigues was of the opinion that even with the improved Cæsarean section, the operation was more fatal to the mother than craniotomy. The chances of saving the mother after craniotomy were five times greater than after Cæsarean section. In certain exceptional cases the latter operation might be deliberately selected, as when the pelvis was generally contracted, rendering extraction of the child after craniotomy exceedingly difficult. As a rule, however, it was better to destroy the child. This was especially true in private practice. The operation of Cæsarean section requires skilled assistance and a qualified abdominal surgeon.

Dr. Lusk said that he agreed thoroughly with the remarks of Dr. Garrigues. We had no moral right to balance one life against another, and decide to kill one human being to save another. His custom was to put the question to the mother and friends, stating without exaggerations the dangers of the different operations, and allow them to decide if they cared to take the extra risk involved in Cæsarean section. The chances of the mother were certainly not so good. In those hospitals in which the mortality from Cæsarean section had been so much reduced, that from craniotomy was almost nothing. The operations could not be compared with an ordinary laparotomy, for the conditions were different. A number of skilled assistants were necessary, while to do a craniotomy one skilled operator was all required.

### A QUESTION IN ETHICS.

At the last meeting of the County Society an interesting discussion occurred over the question of the propriety of physicians allowing themselves to be interviewed by newspaper reporters. Dr. F. R. Sturgis introduced the subject, and took the ground that on matters pertaining to public health, or prominent new methods of treatment, or unusual surgical operations, physicians were justified in responding to the request of newspaper men for an expression of opinion.

Dr. Shrady, editor of the *Medical Record*, believed that when a physician could feel that he was called upon as the spokesman of his profession in conveying to the public useful information, he was perfectly justified in speaking. When, however, he magnified his own acts, and the *ego* appeared in every sentence, it would be better for him and for the profession if he had not spoken.

WM. L. RUSSELL.

151 *East 50th Street.*

---

PROF. WILLIAM ROSE, of London, recently removed the superior maxillary bone and excised the Gasserian ganglion for severe *tic douloureux*. This is said to be the only instance in which this operation has been done. The patient's eye was lost on the affected side, but the neuralgia ceased. *Gaillard's Medical Journal.*

